# Passive leg raise testing effectively reduces fluid administration in septic shock after correction of non-compliance to test results

**DOI:** 10.1186/s13613-016-0225-6

**Published:** 2017-01-03

**Authors:** Arjanne Rameau, Eldert de With, Evert Christiaan Boerma

**Affiliations:** Department of Intensive Care, Medical Centre Leeuwarden, Henrie Dunantweg 2, 8934 AD Leeuwarden, The Netherlands

**Keywords:** Sepsis, Fluid administration, Passive leg raise test, Knowledge-to-care gap, Non-compliance, Implementation

## Abstract

**Background:**

Fluid resuscitation is considered a cornerstone of shock treatment, but recent data have underlined the potential hazards of fluid overload. The passive leg raise (PLR) test has been introduced as one of many strategies to predict ‘fluid responsiveness.’ The use of PLR testing is applicable to a wide range of clinical situations and has the potential to reduce fluid administration, since PLR testing is based upon (reversible) autotransfusion. Despite these theoretical advantages, data on the net effect on fluid balance as a result of PLR testing remain scarce.

**Methods:**

We performed a prospective single-center multi-step interventional study in patients with septic shock to evaluate the effect of implementation of PLR testing on the fluid balance (FB) 48 hours after ICU admission. All patients were equipped with a PiCCO^®^ device for pulse contour analysis to guide fluid administration. An increase in stroke volume (SV) ≥ 10% was considered a positive test result.

**Results:**

Before introduction of PLR testing, 21 patients were prospectively included in period 1 with a median FB of 4.8 [3.3–7.8]L. After an extensive training program, PLR testing was introduced and 20 patients were included in period 2. Median FB was 4.4 [3.3–7.5]L and did not differ from period 1 (*p* = 0.72). Further analysis revealed that non-compliance to the PLR test result was 44%. These findings were discussed with all ICU doctors and nurses. By consensus, non-compliance to the PLR test result was identified as the main reason for unsuccessful implementation of PLR testing. After this evaluation, 19 patients were included in period 3 under equal conditions as in period 2. In this period, median FB was 3.1 [1.5–4.9]L and significantly reduced in comparison with periods 1 and 2 (*p* = 0.016 and *p* = 0.023, respectively). Non-compliance was 9% and significantly lower than in period 2 (*p* = 0.009).

**Conclusion:**

Implementation of PLR testing in patients with septic shock reduced fluid administration in the first 48 hours of ICU admission significantly and substantially. To achieve this endpoint, substantial non-compliance of ICU team members had to be addressed. Fluid administration despite a negative PLR test was the most common form of non-compliance.

**Electronic supplementary material:**

The online version of this article (doi:10.1186/s13613-016-0225-6) contains supplementary material, which is available to authorized users.

## Background

Although fluid administration remains a cornerstone in the resuscitation of shock, recent publications have highlighted the potential hazards of fluid overload. Both in sepsis and in other disease states a positive fluid balance appeared to be independently associated with morbidity and mortality [1–5]. Classically, the evaluation of the effect of fluid administration is based upon the relationship between preload and stroke volume of the left ventricle and referred to as ‘fluid responsiveness’ [6]. In the clinical setting, it is pivotal to develop strategies with adequate positive and negative predictive power to discriminate between responders and non-responders, since it is estimated that only half of the ICU patients is ‘fluid responsive’ [7]. Dynamic indices, based upon the respiratory–circulatory interaction, have the potential to predict the effect of a fluid bolus adequately before the actual administration, but its general applicability is largely hampered by a range of technical limitations [8]. The passive leg raise (PLR) test has been developed as an alternative strategy to predict ‘fluid responsiveness’ without the risk of unwanted irreversible fluid loading [9–11]. The response in stroke volume to a PLR test substantially reflects the subsequent effect of fluid administration, with the potential to reduce unwanted fluid loading [11, 12].

Despite the clear physiological principles of PLR testing, data on the net reduction in fluid balance of critically ill patients remain scarce. The main topic of the vast majority of studies is limited to the sensitivity and specificity of the PLR test in comparison with a well-known standard. However, in the clinical setting correct measurement, correct interpretation and correct application are all needed to change therapeutic behavior of doctors and nurses effectively [13]. This study focusses on the question: Is it possible to implement PLR testing in such manner that it effectively leads to reduction in fluid administration?

## Methods

### Setting, patients and design

This single-center study was carried out in a 20-bed mixed intensive care unit. It was designed as a prospective multi-step intervention study before and after the introduction of the PLR test. All patients ≥18 years were considered eligible for the study if they fulfilled the criteria for septic shock as the main reason for ICU admission and if they were equipped with a PiCCO^®^ device. Exclusion criteria comprised pregnancy, open abdomen, abdominal hypertension, recent head/pelvis/lower extremity trauma and deep venous thrombosis.

### Protocol

#### Period 1

In 2012 consecutive patients who fulfilled the entry criteria were prospectively identified. From our patient data management system (PDMS) demographic data, Acute Physiology And Chronic Health Evaluation (APACHE II) score, source of sepsis and fluid balance 48 h after ICU admission were extracted.

#### Period 2

Immediately after having included the required amount of patients in period 1, an educational program for all ICU doctors and nurses was introduced (Additional file 1). This program included both training in theoretical background and practical aspects of PLR testing. In addition, a flowchart was embedded in our bedside PDMS to identify patients eligible for the study and to provide practical assistance in the execution of the test and interpretation of its results. During inclusion of patients in period 2, ‘super-users’ for floor support were available during all shifts. After completion of the educational program in 2013, an inclusion episode in period 2 started.

#### Period 3

After inclusion of the number of patients needed for period 2, statistical analysis of the data was performed. Results were shared with all ICU doctors and nurses and discussed in detail in small groups. A survey was sent to all members of the ICU team to identify reasons for non-compliance to the study protocol [14]. Subsequently, a team consensus on reasons for non-compliance as well as suggestions for practical solutions was briefed to all personnel. After completion of this evaluation-and-feedback program, inclusion of patients in period 3 was performed in 2015.

### Measurements

**Fig. 1:**
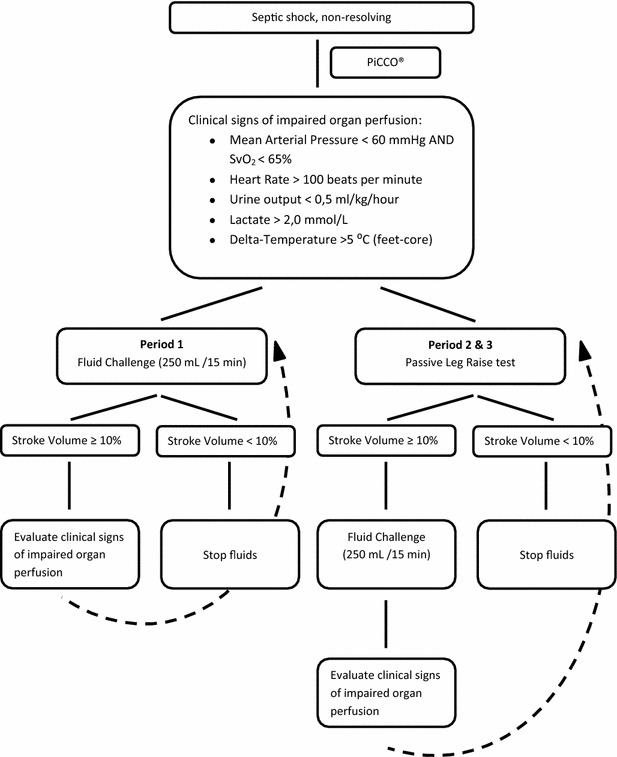
Flowchart PiCCO^®^-guided fluid administration

**Fig. 2:**
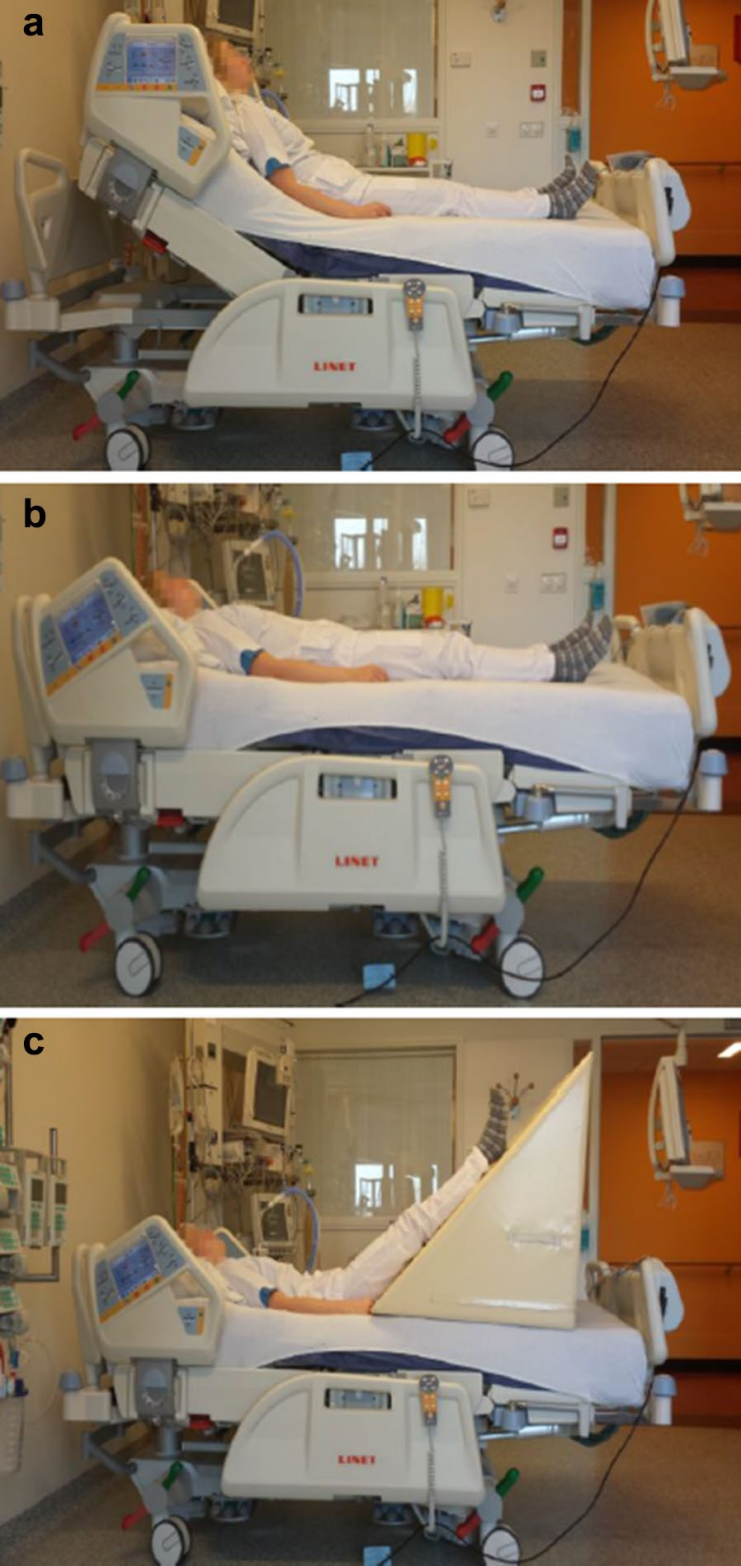
Setting passive leg raise test. **a** Resting condition. **b** Supine position (1 min). **c** Passive leg raise test

During the entire study period all included patients were sedated with midazolam/fentanyl and equipped with an invasive cardiac output device (PiCCO^®^, Maquet, Munich, Germany), enabling the tracing of changes in stroke volume (SV) within the short timeframe of fluid administration and/or the PLR test. Until the PiCCO^®^ device was in place fluid administration was left to the discretion of the attending physician. In period 1 clinical signs of impaired organ perfusion were followed by 250 mL ringer’s lactate administration in 15 min (Fig. 1). Subsequent changes in SV were recorded; an increase in SV < 10% was considered a contraindication for additional fluid administration in the following period. In periods 2 and 3 clinical signs of impaired organ perfusion were followed by PLR testing. After a 1-min adaptation period in a supine position of the patient, a 45° angle of the lower extremities was achieved with a tailor made cushion during 2 min (Fig. 2). An increase in SV ≥ 10% during the PLR test was considered ‘fluid responsive’ [11, 12]. In this case 250 mL ringer’s lactate was administered in 15 min after the PLR test. SV before and after each fluid challenge were registered (Fig. 1). The decision to perform a PLR test and to administer fluids to the patient was ultimately left to the discretion of the attending physician.

### Statistics

Primary endpoint was the difference between groups in fluid balance 48 h after ICU admission. Based upon a preliminary sample, an α of 0.05 and a ß of 0.2 (power 0.8), we anticipated a sample size of 20 patients per group to detect a difference of 1000 mL. One extra patient per group was included to compensate for loss to follow-up. Data are presented as median [IQR]. Applicable tests for independent nonparametrical data were used. A *p* value <0.05 was considered statistically significant.

## Results

### Period 1

Twenty-one patients were included. Baseline characteristics are summarized in Table 1. Median fluid balance 48 h after ICU admission was 4.8 [3.3–7.8]L.

**Table 1:** Baseline characteristics

Variables	Period 1 (*n* = 21)	Period 2 (*n* = 20)	Period 3 (*n* = 19)	*p* value
Men, *n* (%)	10 (48)	10 (50)	12 (63)	0.58
Age (years)	73 [58–79]	66 [56–69]	66 [47–69]	0.14
APACHE II	22 [16–29]	24 [18–32]	22 [16–27]	0.81
Weight (kg)	85 [76–90]	84 [66–104]	83 [75–100]	0.91
Origin of sepsis, *n* (%)				
Respiratory	3 (14)	9 (45)	8 (42)	
Abdominal	16 (76)	10 (50)	10 (53)	
Cutaneous	2 (10)	0 (0)	0 (0)	0.14
Other	0 (0)	1 (5)	1 (5)	
Use of ventilator, *n* (%)	21 (100)	20 (100)	19 (100)	1.00
NE, maximum dose (μg/kg/min)	0.15 [0.1–0.28]	0.16 [0.13–0.28]	0.11 [0.09–0.26]	0.48
Highest lactate (mmol/L)	2.7 [2.1–4.4]	2.9 [2.0–3.8]	2.1 [1.7–3.8]	0.22
Fluids before 1st PLR test (L)	–	2.1 [1.0–3.7]	2.3 [0.9–3.2]	0.81

Period 1 controls, period 2 intervention before feedback, period 3 intervention after feedback. Data are presented as median [IQR] or as numbers (%)

*APACHE* Acute Physiology And Chronic Health Evaluation, *NE* norepinephrine, *PLR* passive leg raise

### Period 2

**Fig. 3:**
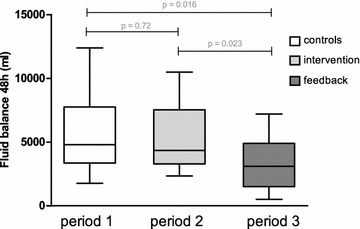
Primary endpoint: fluid balance 48 h after ICU admission

**Fig. 4:**
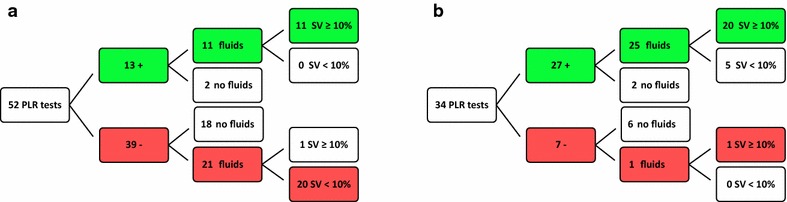
Overview of passive leg raise test results and subsequent fluid administration. **a** Period 2, **b** Period 3

After the initial training program 21 patients were included; one patient was excluded due to extreme intestinal fluid loss. Baseline characteristics are summarized in Table 1. Median fluid balance 48 h after ICU admission was 4.4 [3.3–7.5]L and did not significantly differ in comparison with patients during period 1 (*p* = 0.72, Fig. 3). During period 2 a total of 52 PLR tests were performed in the first 48 h of ICU admission. Thirteen PLR tests (25%) were positive, not followed by fluid administration in two cases (non-compliance 2/13, 15%). In the remaining 11/13 (85%) cases a positive PLR test was followed by fluid administration, resulting in an increase of SV ≥ 10% in all cases (Fig. 3). Thirty-nine PLR tests were negative, followed by fluid administration in 21 cases (non-compliance 21/39, 54%). After fluid administration an increase of SV ≥ 10% was observed in only 1 out of 21 cases (5%, Fig. 4).

After feedback of these data to the doctors and nurses, the ICU team by consensus designated non-compliance to a negative test result (i.e., to administer fluids after a negative PLR test) as the main reason for failure of successful implementation of PLR testing. All doctors and nurses who did not comply to the negative PLR test result declared that their judgement to administer fluids despite the negative test result was based on ‘gut feeling’ and not on the bases of specific hemodynamic variables. According to the team survey the average number of PLR tests per included patient (2.7 ± 1.7) was mainly limited due to impracticalities related to the PLR cushion, such as size, weight and cleaning. Since these practicalities were difficult to overcome within a reasonable timeframe, it was decided to start period 3 without changes in the way PLR testing was executed.

### Period 3

After feedback of data from period 2 to the ICU team and the subsequent analysis of the main reason for non-compliance, 21 patients were included in period 3, under otherwise equal conditions as in period 2. Two patients were excluded due to correction of the initial diagnose of ICU admission. Baseline characteristics are summarized in Table 1 and did not significantly differ across groups. Median fluid balance 48 h after ICU admission was 3.1 [1.5–4.9]L and was significantly different from patients in periods 1 and 2 (*p* = 0.016 and *p* = 0.023, respectively; Fig. 3). During period 3 a total of 34 PLR tests were performed in the first 48 h of ICU admission. Twenty-seven PLR tests (79%) were positive, not followed by fluid administration in two cases (non-compliance 2/27, 7%). In the remaining 25/27 (93%) cases a positive PLR test was followed by fluid administration, resulting in an increase of SV ≥ 10% in 20/25 (80%) cases (Fig. 4). Seven PLR tests were negative, followed by fluid administration in one case (non-compliance 1/7, 14%). Overall, non-compliance (i.e., fluid administration after a negative PLR test plus no fluid administration after a positive PLR test, respectively) reduced significantly between periods 2 and 3 (44 vs. 9%, *p* = 0.001).

## Discussion

This study shows that successful implementation of PLR testing effectively reduces fluid administration. However, introduction of a seemingly simple intervention, based on solid scientific principles, does not automatically guarantee improvement in a clinically relevant endpoint. Despite an intensive training program the fluid balance after 48 h of ICU admission did not change in patients with septic shock. Only after the ICU team became aware that non-compliance to the test results was the main reason for the failure of successful implementation of PLR testing, the administered amount of fluids in the first 48 h of ICU admission reduced significantly and substantially.

These data are in line with the literature. The recent FENICE trial not only revealed that doctors use a wide range of hemodynamic variables to assess ‘fluid responsiveness,’ including endpoints that have been unmasked in the medical literature as erroneous [15]. Moreover, the percentage of patients receiving fluid administration did not differ between responders and non-responders. In other words, the test results did not seem to influence the decision to give fluids. These data reflect a broader challenge in (critical care) medicine, referred to as the ‘knowledge-to-care gap’ [16]. Reasons for unsuccessful or non-implementation of guidelines in clinical practice are now being recognized beyond the lack of knowledge, including (lack of) comprehensiveness, relevance, simplicity, applicability and presence of (negative) role models [14]. Our strategy of self-assessment of the ICU team after the failed attempt to implement PLR testing (period 2) may be referred as a form of ‘participatory action research,’ initially implemented in community health medicine [17]. Such form of participation may also be transformed to involvement of ICU personnel in the analysis of reasons for ‘knowledge-to-care gaps’ and potential solutions [18]. Ideally, this is an ongoing process, since reassessment of compliance to guidelines over longer periods of time than observed in our study appears to be important as well [19].

Our study has potential limitations. Results of this single-center study in patients with septic shock may not be applicable to all settings and patient categories. Small groups and the inevitable evaluation over time between the three periods carry the potential of unrecognized biases. Rather, this study may serve as an example to raise attention to the ‘knowledge-to-care gap’ and potential solutions. Even a simple, solid and cheap intervention, such as PLR testing, needs a careful implementation strategy that includes feedback of predefined output to become clinically successful. The maximum effect of PLR testing in our setting may have been underestimated. Clearly, the limited number of PLR tests in periods 2 and 3 deserve further investigation and may reflect another form of non-compliance. In addition, the way PLR testing and subsequent fluid administration was performed may have influenced the results. Autotransfusion by means of backward tilting from a semi-recumbent position may influence the predictive value of the test to some extent [20]. Nevertheless, during period 2 in our study the positive and negative predictive value of the PLR test was excellent.

The effect of PLR testing on the use of fluids in our ICU may have been either a direct or indirect effect. It is of note that in period 3, after feedback and assessment of reasons for non-compliance the number of positive PLR test was considerably higher than in period 2. This may simply be coincidence, but alternately reflect a different awareness and timing of ICU team members with respect to fluid administration. Whether or not a reduction in fluid balance 48 h after ICU admission leads to a reduction in mortality was outside the scope of the study.

## Conclusions

Implementation of PLR testing in patients with septic shock reduced fluid administration in the first 48 h of ICU admission significantly and substantially. To achieve this endpoint considerable non-compliance of ICU team members had to be addressed. Fluid administration despite a negative PLR test was the most common form of non-compliance.

## Additional file

**Additional file 1.** Educational training program.

## Abbreviations

ICU: intensive care unit
PLR: passive leg raise
PDMS: patient data management system
APACHE: Acute Physiology And Chronic Health Evaluation
SV: stroke volume
