# Supplementary material for: Passive leg raise testing effectively reduces fluid administration in septic shock after correction of non-compliance to test results
Source: Ann Intensive Care. 2017 Jan 3;7:2. doi: 10.1186/s13613-016-0225-6 (PMC5209308; doi:10.1186/s13613-016-0225-6)
Supplement: Supplementary file 1 — Additional file 1. Educational training program. [file 13613_2016_225_MOESM1_ESM.docx]

**Table. Content education session**

1.Classical educational group sessions for doctors and nurses ‘why and how to perform PLR tests’

2.Classical educational group sessions for doctors and nurses about the research protocol

3. Individual bedside teaching for doctors and nurses how to perform and interpret PLR tests

4. Instruction sheets in patient data management system (PDMS)

5. Pop ups in PDMS to alert staff that a specific patient is potentially eligible for the study

6. Pop ups in PDMS to alert staff that the patient is a ‘non-responder’ or a ‘responder’ after PLR tests and/or fluid administration

7. Super-users during all shifts as floor support for doctors and nurses

8. Classical educational group sessions for doctors to give feedback on the results of period 2 plus structured discussion why and how to improve protocol compliance

9. Classical educational group sessions for nurses to give feedback on the results of period 2 plus structured discussion why and how to improve protocol compliance

10. Survey for doctors and nurses to explore reasons for non-compliance to test results

11. Multiple reminders in weekly ‘ICU newsletter’
